# Supplementary material for: The Geomagnetic Field (GMF) Modulates Nutrient Status and Lipid Metabolism during Arabidopsis thaliana Plant Development
Source: Plants (Basel). 2020 Dec 8;9(12):1729. doi: 10.3390/plants9121729 (PMC7762565; doi:10.3390/plants9121729)
Supplement: Supplementary file 1 [file plants-09-01729-s001.pdf]

ROSETTE STAGE OF DEVELOPMENT

15 days
GMF

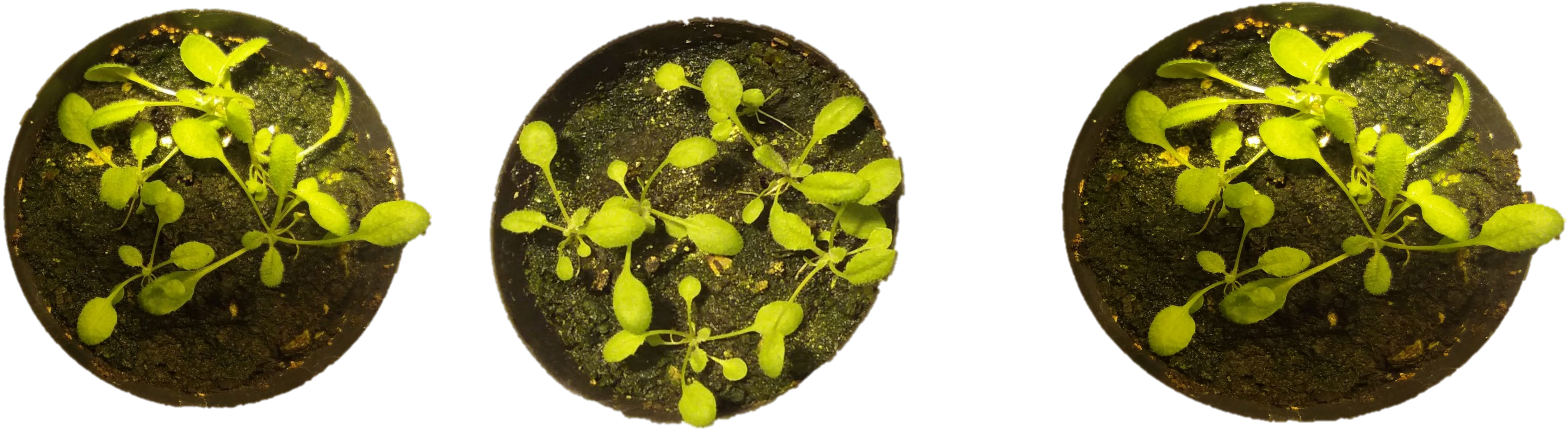

15 days
NNMF

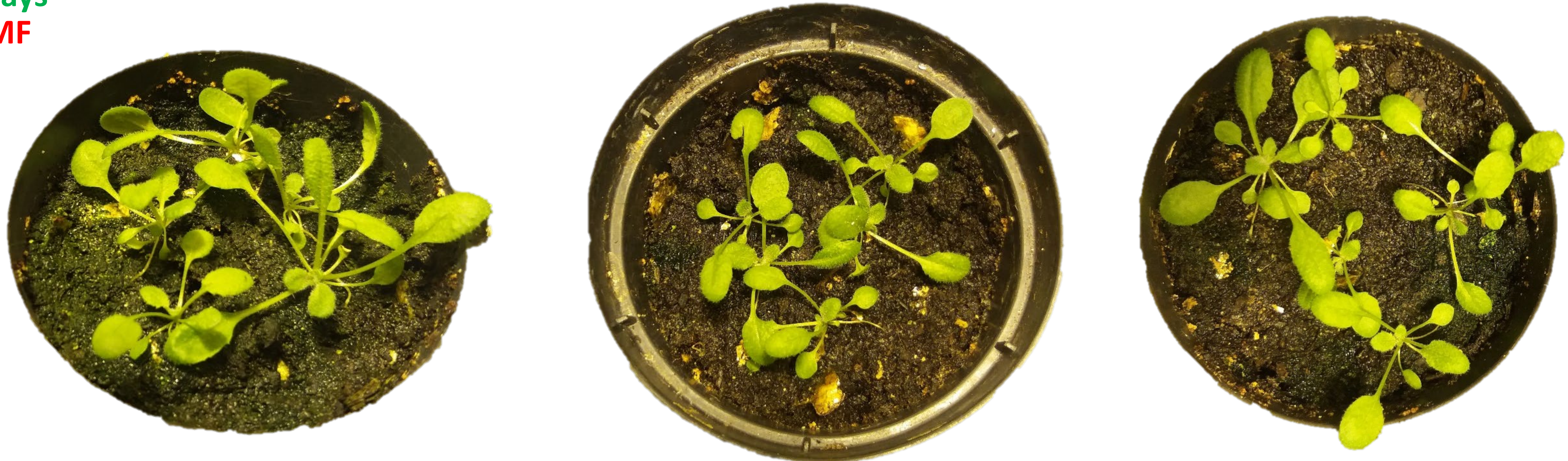

BOLTING STAGE OF DEVELOPMENT

21 days
GMF

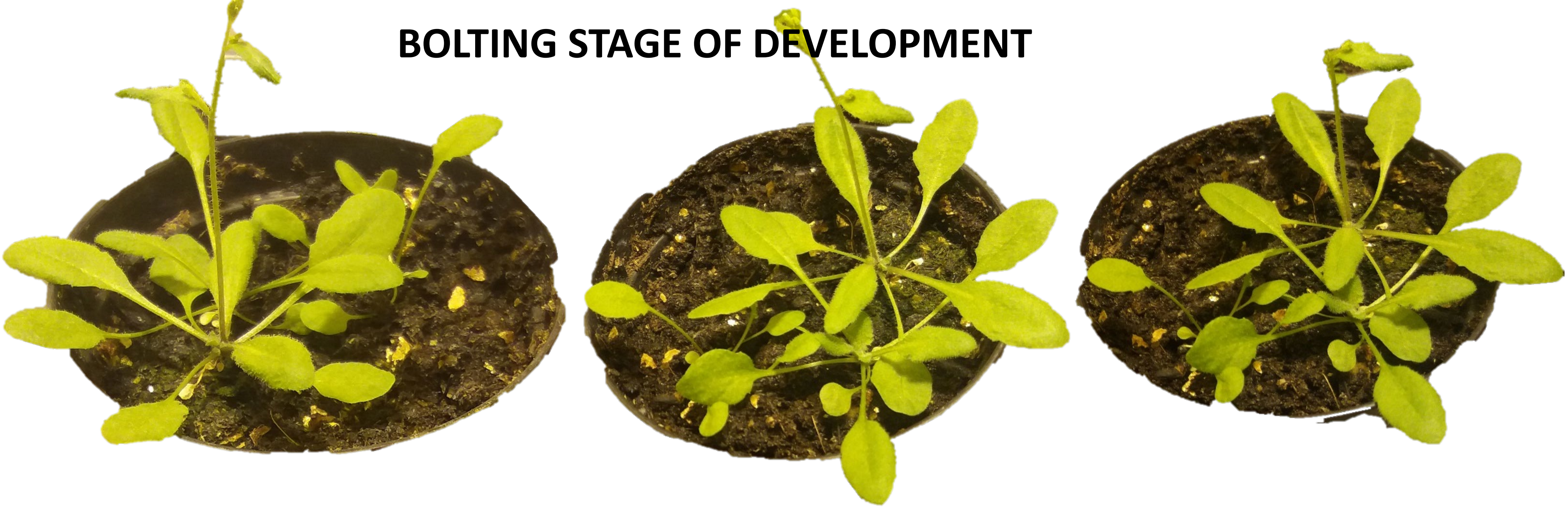

21 days
NNMF

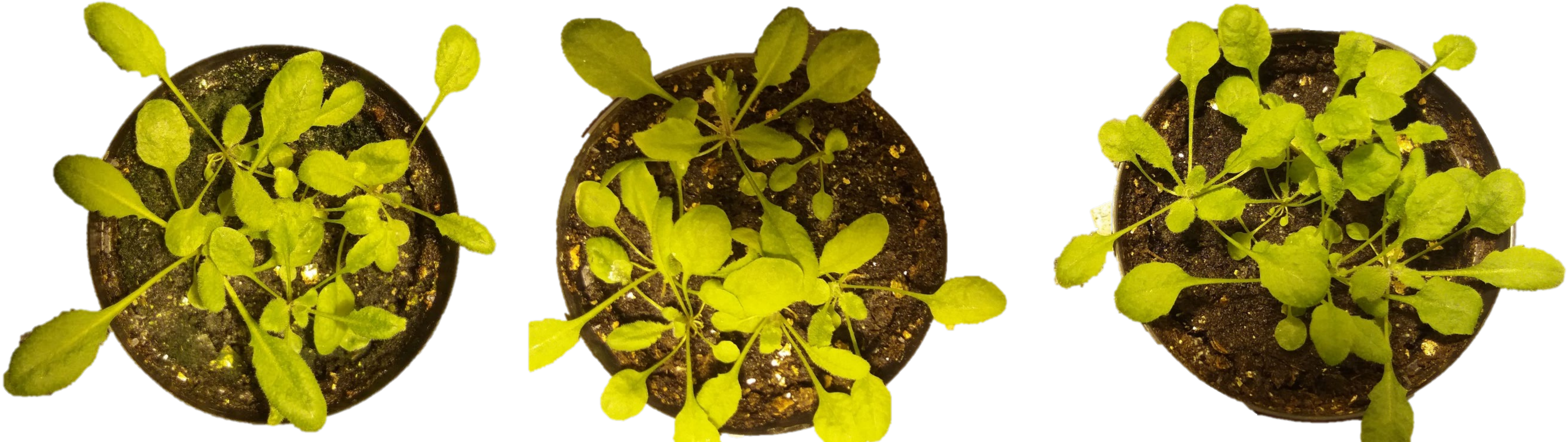

FLOWERING STAGE OF DEVELOPMENT

30 days
GMF

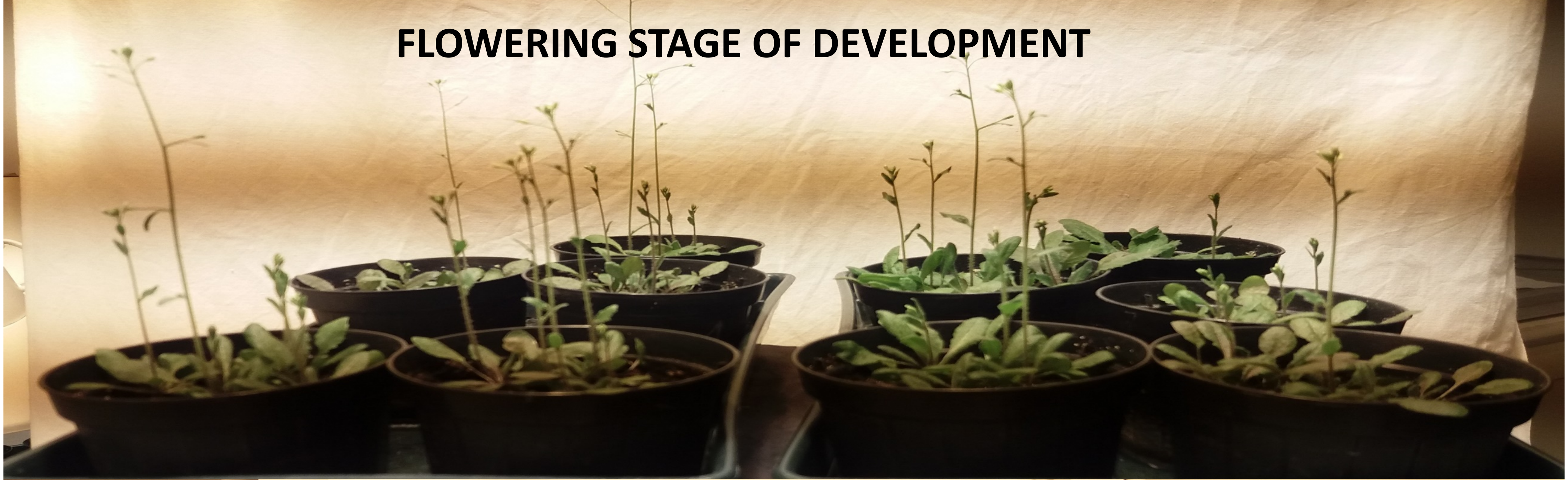

30 days
NNMF

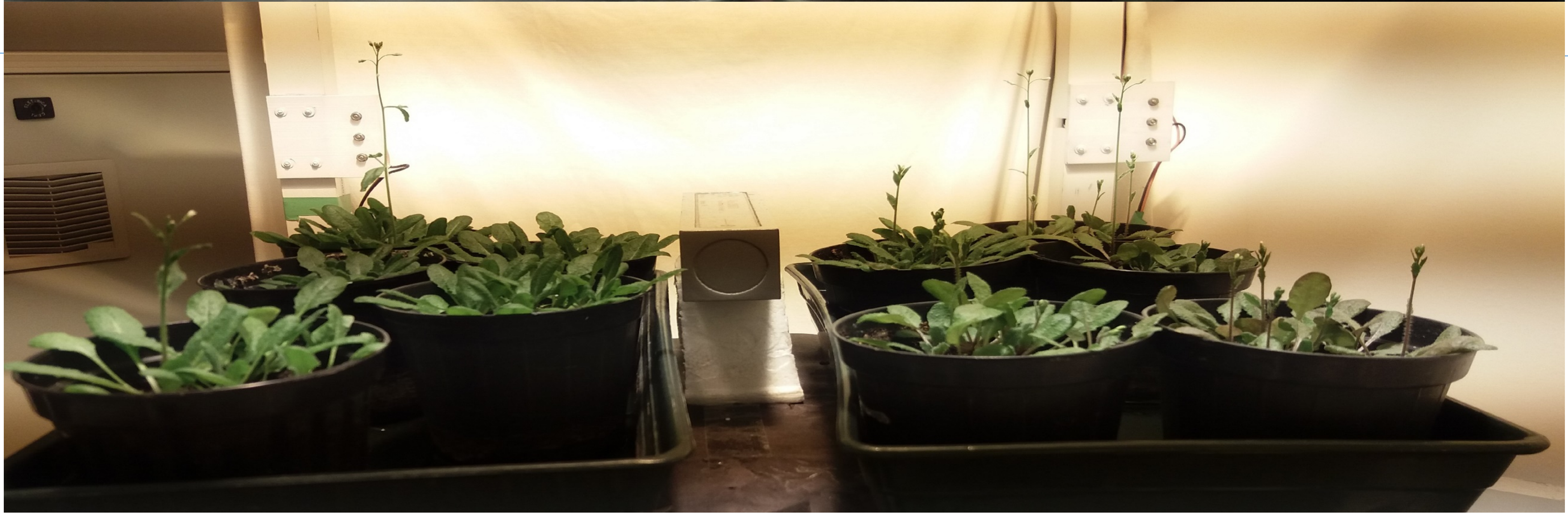

SEED-SET STAGE OF DEVELOPMENT

35 days
GMF

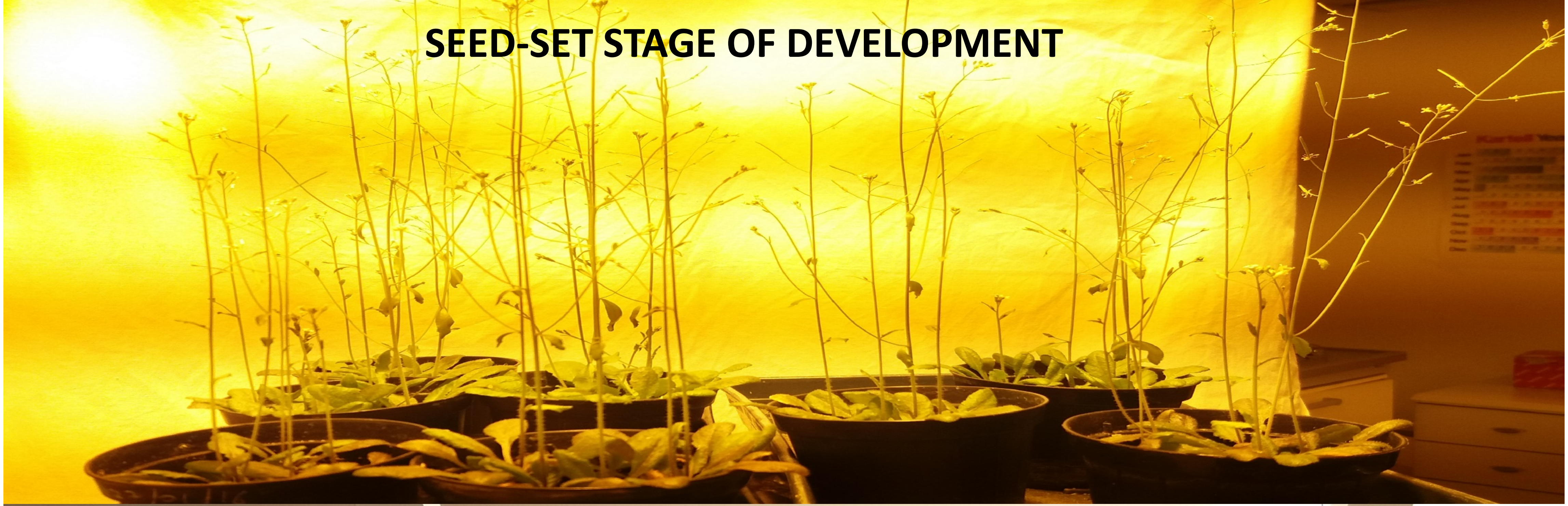

35 days
NNMF

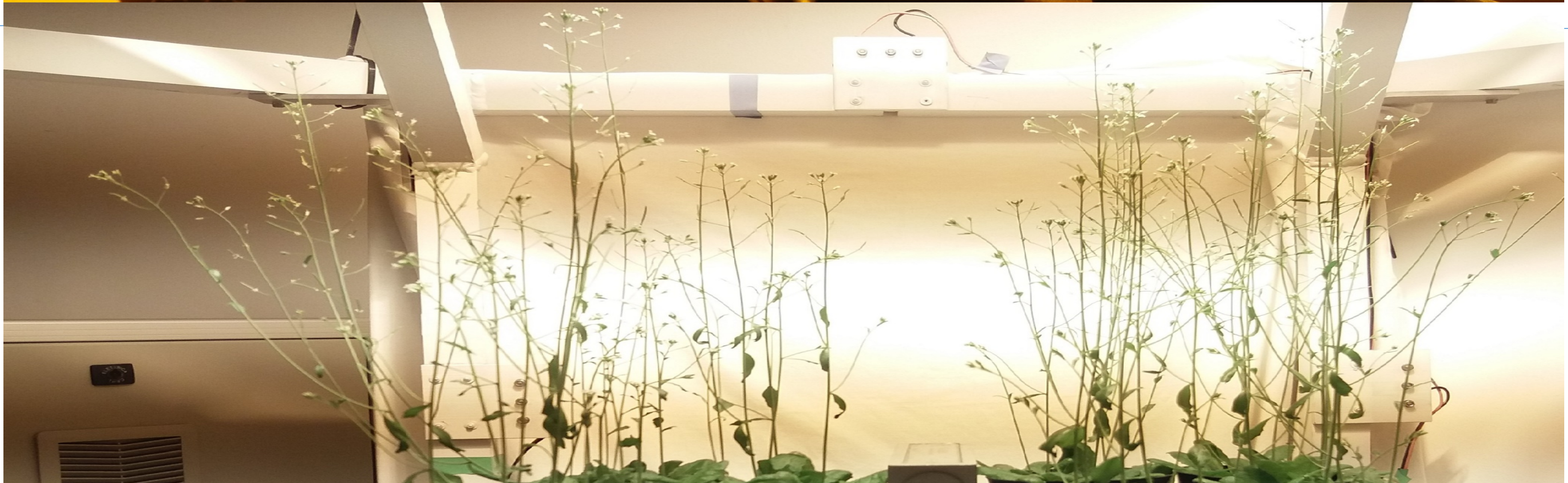

Supplementary Table S1. Direct comparison of morphological and developmental parameters between Arabidopsis thaliana exposed to either GMF or NNMF. Data are expressed as means (± standard deviation) from three independent biological replications. Boldfaced figures indicate significant (P < 0.01) differences between GMF and NNMF.

Parameters	Rosette		Bolting		Flowering		Seed-set	
	GMF	NNMF	GMF	NNMF	GMF	NNMF	GMF	NNMF
Leaf area index (LAI) (cm)	12.93 ± 0.46	13.34 ± 0.23	22.72 ± 0.38	21.79 ± 0.57	**63.18 ± 1.07**	**56.63 ± 0.67****	**109.81 ± 0.61**	**105.58 ± 0.75****
Stem Length (cm)	1.07 ± 0.25	0.73 ± 0.15	**8.02 ± 0.08**	**4.85 ± 0.24****	**13.28 ± 0.43**	**7.61 ± 0.49****	**19.13 ± 0.24**	**12.54 ± 0.58****
Shoot Fresh weight(mg/plant)	0.54 ± 0.02	0.51 ± 0.02	**2.87 ± 0.01**	**2.50 ± 0.04****	**25.89 ± 0.02**	**19.96 ± 0.10****	**36.24 ± 0.05**	**33.10 ± 0.05****
Shoot Dry weight (mg/plant)	0.05 ± 0.00	0.05 ± 0.00	**0.29 ± 0.00**	**0.25 ± 0.00****	**2.78 ± 0.00**	**2.15 ± 0.01****	**3.62 ± 0.00**	**2.76 ± 0.00****

Supplementary Table S2

Primers used in quantitative real time PCR experiments

Gene Code	Gene	Forward primer (5’-3’)	Reverse primer (5’-3’)
**Reference genes**			
At2g37620	*ACT1*	TGCACTTCCACATGCTATCC	GAGCTGGTTTTGGCTGTCTC
At5g19510	*eEF1Balpha2*	ACTTGTACCAGTTGGTTATGGG	CTGGATGTACTCGTTGTTAGGC
At1g13440	*GAPC2*	TCAGGAACCCTGAGGACATC	CGTTGACACCAACAACGAAC
At1g51710	*UBP6*	GAAAGTGGATTACCCGCTG	CTCTAAGTTTCTGGCGAGGAG
**Target GENES**			
At1g01120	*KCS1*	GGTTAAAGCTGGTGACCGAC	TCATCTCCTCCGTCGAAACC
At1g25450	*KCS5*	GCTACAAACCTCCTGTCACG	CCTGATCGCTCGAGGATTCT
At1g68530	*KCS6*	CAGCACGGAGATCATAACGC	CGGCGGTTTGACATGTGTAT
At2g15090	*KCS8*	AAGCAGGCATGTGTTGGAAG	AGTGGGAAGCACTGAAGACC
At2g28630	*KCS12*	GTTGCATCCAAGCCGAAGAT	ACGGGAAGAATCTTGGGTGT
At1g06350	*ADS4*	GACATGGCTCGTAAACTCGG	CGATGACTCAAACGCGTGAT
At2g15230	*LIP1*	GCCTCACCCATCATCTGTCA	TTCGACAGCCCATAAGTCCG
At1g27950	*LTPG1*	CAAGCGAAGACAGGAGGACA	TGGCGAGCTAGGTGAAATCC
